# Cellular Senescence in Traumatic Brain Injury: Evidence and Perspectives

**DOI:** 10.3389/fnagi.2021.742632

**Published:** 2021-09-28

**Authors:** Nicole Schwab, Emily Leung, Lili-Naz Hazrati

**Affiliations:** ^1^Department of Laboratory Medicine and Pathobiology, University of Toronto, Toronto, ON, Canada; ^2^The Hospital for Sick Children, Toronto, ON, Canada

**Keywords:** cellular senescence, mild traumatic brain injury, concussion, brain trauma, chronic traumatic encephalopathy

## Abstract

Mild traumatic brain injury (mTBI) can lead to long-term neurological dysfunction and increase one's risk of neurodegenerative disease. Several repercussions of mTBI have been identified and well-studied, including neuroinflammation, gliosis, microgliosis, excitotoxicity, and proteinopathy – however the pathophysiological mechanisms activating these pathways after mTBI remains controversial and unclear. Emerging research suggests DNA damage-induced cellular senescence as a possible driver of mTBI-related sequalae. Cellular senescence is a state of chronic cell-cycle arrest and inflammation associated with physiological aging, mood disorders, dementia, and various neurodegenerative pathologies. This narrative review evaluates the existing studies which identify DNA damage or cellular senescence after TBI (including mild, moderate, and severe TBI) in both experimental animal models and human studies, and outlines how cellular senescence may functionally explain both the molecular and clinical manifestations of TBI. Studies on this subject clearly show accumulation of various forms of DNA damage (including oxidative damage, single-strand breaks, and double-strand breaks) and senescent cells after TBI, and indicate that cellular senescence may be an early event after TBI. Further studies are required to understand the role of sex, cell-type specific mechanisms, and temporal patterns, as senescence may be a pathway of interest to target for therapeutic purposes including prognosis and treatment.

## Introduction

Mild traumatic brain injury (mTBI) occurs frequently in the general population and includes concussions and subconcussive hits to the head (Carrol et al., 2004). Typically, patients recover within weeks of the injury, however a subset continues to have long-term, persistent symptoms lasting longer than 3 months post-injury, and even years later (Hiploylee et al., 2017). These symptoms vary in nature and severity, but often include anxiety, depression, sleep disturbances, light sensitivity, headaches, fatigue, behavioral abnormalities, and memory and attention problems (Ryu et al., 2009). In the long-term, people who have a history of mTBI are at a higher risk of developing several neurodegenerative diseases, including “classic” types such as Alzheimer's disease (AD) (Mortimer et al., 1991) and Parkinson's disease (PD) (Jafari et al., 2013), but also more recently mTBI has been specifically linked to chronic traumatic encephalopathy (CTE) (Stein et al., 2014; Maroon et al., 2015; McKee et al., 2016). It is currently unclear what pathophysiological mechanisms drive the short- and long-term symptoms of mTBI and, by extension, the mechanism by which mTBI primes the brain toward these neurodegenerative disorders is not well-established.

Recent studies have identified stress-induced cellular senescence, specifically DNA damage induced, as a possible mechanism at play following mTBI. Various kinds of DNA damage exist, including single-stranded breaks (SSBs), double-stranded breaks (DSBs), and oxidative damage (Cadet and Davies, 2017) which can come from both endogenous metabolic sources as well as exogenous stress or toxins. Cell's response to DNA damage, appropriately named the DNA damage response (DDR), exists to counteract these genotoxic events and restore DNA integrity. The DDR consists of a large number of enzymes, transcription factors, and signaling molecules which all play crucial roles in maintaining DNA structure (Li et al., 2016). For example, in response to DSBs, histone family member X (H2AX) is phosphorylated at Ser139 by ataxia telangiectasia mutated (ATM), a sensor in this pathway. H2AX therefore becomes γH2AX, which is considered a robust marker of DSBs (Burma et al., 2001). This phosphorylation step leads to the recruitment of downstream repair proteins, such as RAD51 and BRCA1, which may result in efficient repair of the break (Stolz et al., 2011). However, if DNA damage is persistent or overwhelming to the cell, the repair may become inefficient. Indeed, deficient DNA repair is considered a hallmark of aging (Garinis et al., 2008), neurodegenerative disease (Madabhushi et al., 2014), depression (Borgesius et al., 2011), and cognitive decline (Simpson et al., 2015).

When DNA repair becomes inefficient, DNA damage results in the activation of downstream senescence pathways including induction of cell-cycle arrest through cyclin-dependent kinase inhibitor 2A (p16) (Rayess et al., 2012), cyclin-dependent kinase inhibitor 1A (p21) (Yosef et al., 2017), or Transformation related protein 53 (p53)-dependent (Rufini et al., 2013) pathways, transcription of pro-inflammatory senescence-associated secretory phenotype (SASP) factors (Hiploylee et al., 2017) and reduced transcriptional expression of DNA repair enzymes such as BRCA1 and EXO1 (Collin et al., 2018). Indeed, senescence is a state of permanent cell-cycle arrest, chronic inflammation, and dysfunction (Di Micco et al., 2021). The SASP consists of secreted interleukins, cytokines, chemokines, and matrix metalloproteinases (Freund et al., 2010), which can reinforce the cell's senescent phenotype while promoting paracrine effects to neighboring cells. Several methods are used to identify senescent cells in the brain, including gene expression changes consistent with cell-cycle arrest (expression of p16, p21, or p53-dependent pathways) and SASP, elevated expression of senescence-associated beta-galactosidase (SA-β-Gal) on the tissue (Dimri et al., 1995), morphological changes such as cytoplasmic swelling (Dou et al., 2017), and changes to nuclear markers [loss of H3K27Me3 expression (Ito et al., 2018) and loss of Lamin B1 expression (Freund et al., 2012)], and subsequent rearrangement of chromatin resulting in senescence-associated heterochromatic foci seen on tissue (Aird and Zhang, 2013). It is also important to note in brain tissue that neuronal senescence remains a topic of controversy with some researchers referring to neuronal senescence as “senescence-like,” as post-mitotic cells do not regularly undergo a cell cycle (Fielder et al., 2017; Moreno-Blas et al., 2019). Glial cell senescence is better established (Chinta et al., 2013; Cohen and Torres, 2019; Han et al., 2020) and is typically assessed based on the assays mentioned here. However, it is important to note that the functional repercussions of these changes in astrocytes remain elusive, as astrocytes rarely divide in healthy tissue and could therefore also be considered post-mitotic (Escartin et al., 2021).

The effects of senescent glial cells on overall brain health are immense. Depending on the cell type affected, functional changes can differ. For example, senescent astrocytes can result in loss of neuronal trophic support (Pertusa et al., 2007) and subsequent excitotoxicity (Limbad et al., 2020) whereas senescent oligodendrocytes can result in loss of myelination and loss of axonal health (Tse and Herrup, 2017). In each case, neuronal health is directly affected by glial cell senescence which can greatly impact network activity and neuronal connections (Soto-Gamez et al., 2019). Microglia that become senescent may lose clearance functions (Conde and Streit, 2006) or increase levels of inflammation in the brain (Koellhoffer et al., 2017), vastly altering the brain's immune system. Thus, accumulation of senescent glial cells likely has implications for widespread tissue dysfunction beyond the immediate area of initial damage. Further, there is an established connection between senescent brain cells and both tau, a microtubule stabilizing protein which form hyperphosphorylated aggregates in a diseased state, and β-amyloid, a product of amyloid precursor protein which forms neurotoxic aggregates, with cognitive decline (Bussian et al., 2018), suggesting this could be the basis of several neurological sequalae in conditions like brain trauma.

This narrative review will first outline the clinical and molecular aspects of mTBI, and will then review the existing evidence supporting DNA damage and cellular senescence as possible drivers of these phenotypes.

## Context: mTBI and its Clinical Manifestations

Traumatic brain injury (TBI) is a top cause of disabilities and death globally, with an estimated 10 million individuals affected yearly (Hyder et al., 2007). Of these injuries, mTBI is the most prominent although its exact incidence is difficult to discern due to many patients not seeking medical attention (Setnik and Bazarian, 2007). mTBI is common in the general population, most notably as a result of motor vehicle accidents, workplace accidents, and falls, but is even more common in several subpopulations including professional sports players, survivors of domestic violence, and military personnel. These subpopulations further contribute to the underreported incidence of mTBI, as they may not want to be removed from playing games, fear of repercussions from a violent partner, or may not have resources to report and be out of work.

The Glasgow Coma Scale (GCS) is used to classify injuries as mild (score of 14–15), moderate (9–13), or severe (3–8) based on visual, verbal, and motor responses (Teasdale and Jennett, 1974). The lowest score, 3, indicates a deep coma or brain-dead state, and the highest score, 15, indicates an awake fully functional patient. A patient with mild traumatic brain injury may experience a loss of consciousness (LOC) for <30 min (although LOC is uncommon with mTBI), alterations of consciousness for up to 24 h, and post-traumatic amnesia for up to 24 h (Blyth and Bazarian, 2010). Typically, imaging tests such as CT scans and MRIs are not performed for patients suspected of having mTBI because imaging results are normal for mTBI patients and no detectable structural changes occur with this level of injury, including various forms of hemorrhage (Lee and Newberg, 2005).

Throughout the decades it was thought that concussions had little to no impact on brain health, but we now know that this is untrue. mTBI leads to a wide array of neurological symptoms in the immediate time following injury including somatic symptoms (Wäljas et al., 2015) (headaches, fatigue, light sensitivity, balance problems, nausea), cognitive symptoms (memory and attention problems) (McInnes et al., 2017), emotional symptoms (irritability, depression, anxiety) (Max et al., 2012), and sleep abnormalities (Sullivan et al., 2015). The type, severity, and duration of symptoms vary between individuals, but most patients tend to recover within days or weeks of the injury. Approximately 20% of patients who do not recover experience post-concussion syndrome (PCS), defined as individuals who do not recover from concussion symptoms within 3 months (Hiploylee et al., 2017). The first longitudinal study of 110 patients with clinically diagnosed PCS found that although 67% of patients recovered from PCS within the first year and 27% recovered eventually, none of the patients who experienced PCS longer than 3 years recovered at all (Hiploylee et al., 2017). This finding suggests that early treatment of mTBI and PCS are critical for neurological recovery, as pathways that eventually lead to permanent changes in the brain, such as cellular senescence, may be activated.

In the long-term, a history of mTBI is a risk factor for the diagnosis of several neurodegenerative diseases and dementia. Indeed, a large retrospective cohort study of over 800,000 patients found that at least one mTBI in life is associated with a 70% increased risk of early-onset dementia (Barnes et al., 2018). Other epidemiological studies have shown a strong association between mTBI and AD after controlling for family history risk of dementia [Mortimer et al., 1991), and TBI patients have been found to be more likely diagnosed with PD (Gardner et al., 2015), as well as frontotemporal dementia (FTD) (Deutsch et al., 2015). Along with increasing risk of a neurodegenerative disease diagnosis, a longitudinal study of over 600 male and female patients with or without TBI history showed that TBI patients have an earlier onset of clinical diagnosis of several neurodegenerative diseases including AD, dementia with Lewy bodies, progressive supranuclear palsy, corticobasal degeneration, frontotemporal dementia, vascular dementia, and PD as well as mild cognitive impairment (Iacono et al., 2021). In addition, this study showed an earlier age of onset of cognitive decline for TBI patients compared to non-TBI patients, and increased frequency of neuropsychiatric symptoms (Iacono et al., 2021). The authors point out that TBI could be considered an “age-lowering” factor in the onset of cognitive decline and neurodegenerative diseases, independent of other contributing factors such as age, education, or underlying clinical diagnoses (Iacono et al., 2021). In addition to human epidemiology data, animal experiments have given insight into this link. For example, a controlled cortical impact (CCI) model in mice results in extensive neurodegeneration (Hall et al., 2005) in the frontal cortex, hippocampus, corpus callosum, and thalamus. Furthermore, several mouse experiments with human transgenic tau mouse models have revealed that mild injury increases levels of hyperphosphorylated tau (p-tau) (Ojo et al., 2013), the pathological protein which accumulates in both AD and CTE, among other tauopathies. CTE is a neurodegenerative disease caused by head trauma, defined pathologically by the presence of p-tau in both neurons and astrocytes, specifically in the depths of cortical sulci near microvasculature (McKee et al., 2016). CTE is a pathological diagnosis defined at autopsy and is associated with a wide array of neurological symptoms ranging from depression and irritability to severe dementia (Schwab et al., 2021a, Mez et al., 2015).

Overall, the clinical aspects of mTBI are heterogeneous and difficult to predict. Patients may have minimal symptoms and recover quickly, some may experience PCS and recover over time, and others may have long-term neurological problems, develop dementia, or be diagnosed with a neurodegenerative disease at autopsy. Several of these clinical phenotypes including depression (Diniz et al., 2017), anxiety (Ogrodnik et al., 2019), sleep disturbances (Carroll et al., 2016), dementia (Baker and Petersen, 2018), and neurodegeneration (Martínez-Cué and Rueda, 2020) are associated with elevated levels of senescent cells in the brain. Therefore, subsequent sections will summarize the evidence showing accumulation of DNA damage and senescent cells after mTBI, and how known molecular changes in mTBI may in fact be downstream repercussions of stress-induced senescence.

## Evidence of DNA Damage in the Injured Brain

### DNA Damage and Repair in TBI

Several studies have identified increased levels of DNA fragmentation in various models of brain injury (Morita-Fujimura et al., 1999; Zhang et al., 2002; Wang et al., 2014), identifying genotoxic stress as a critical component of the brain's response to trauma. In 2001, a study on rats with moderate TBI using the controlled cortical impact model showed elevation of both double and single-strand DNA breaks (DSBs and SSBs, respectively) in the ipsilateral hemisphere 24 h following injury (Clark et al., 2001). The researchers used several methods to identify DNA damage, including DNA polymerase I-mediated biotin-dATP nick-translation (PANT) for SSB detection, the Klenow fragment of DNA polymerase I-mediated biotin-dATP nick-end labeling (Klenow) for both SSBs and DSBs, and terminal deoxynucleotide transferase-mediated dUTP nick-end labeling (TUNEL) for DSBs (which are usually associated with cell death) (Clark et al., 2001). By 6 h post-injury, most nuclei of cells in the ipsilateral dentate gyrus and cortex were diffusely labeled for both SSBs and DSBs, and by 24 h post-injury DNA damage labeling with both PANT, Klenow, and TUNEL was maximal. However, by 72 h post-injury, cell nuclei showed reduced DNA damage compared to earlier timepoints. At all timepoints studied, no DNA breaks were found in the naïve rats, nor in the contralateral hemisphere. When the researchers double labeled with the neuronal marker NeuN they found that most PANT-positive cells were also NeuN-positive, suggesting a predominance of SSBs in neurons (Clark et al., 2001). Using the same controlled cortical impact model in rats but with a severe form of TBI, another research group identified elevation of 8-hydroxy-2′-deoxyguanosine (8-OHdG), a marker of oxidative DNA damage, in the ipsilateral cortex up to 4 h post-injury (Mendez et al., 2004). Similarly, they found elevation of oxidative DNA damage in the ipsilateral cortex but not in the contralateral cortex of injured animals, and no reactivity was seen in sham animals. Nuclei of both neurons and astrocytes showed reactivity for 8-OHdG, and diffuse cytosolic staining was seen suggesting the presence of mitochondrial DNA damage in addition to nuclear DNA damage (Mendez et al., 2004). In mice using a controlled impact model of mild traumatic brain injury (skull intact), our lab has further identified evidence of the DNA damage response at 24 h post-injury consistent with repair of double-strand DNA breaks, inhibition of cell death pathways, and metabolic changes (Schwab et al., 2021b). In this study, gene expression analysis with NanoString was used to assess the DNA damage response 24 h post-injury in the ipsilateral cortex, and indeed gene set enrichment analysis (GSEA) revealed upregulation of DNA repair pathways particularly toward DSBs, SSBs, D-Loop structures, and oxidative damage, along with cell cycle checkpoints, indicating the activation of the DNA damage response (DDR) and DNA damage (Schwab et al., 2021b). Additional evidence supporting the role of DNA damage in the pathophysiological response to brain trauma has come from knocking down DNA repair factors. Indeed, mice with the DNA repair gene XPA knocked out present with delayed neurobehavioural recovery using the CCI model (Tomasevic et al., 2012). Similarly, cold injury-induced trauma results in reduced expression of the DNA repair factor XRCC 4 h post-injury in mice (Fujimura et al., 2000). Taken together, studies using animal models have strongly shown DNA damage, including SSBs, DSBs, and oxidative damage, to be an aftermath of mild, moderate, and severe TBI.

### DNA Damage in Post-Mortem Brains With History of Trauma

While animal models of TBI are vital for understanding pathophysiological mechanisms, they have clear limitations for clinical translation. It is therefore critical to study both pre- and post-mortem human patients in order to understand how molecular findings in animal models may translate to real life, and to develop therapeutic strategies. Brain banks for studying TBI are growing and are often used to study pathological consequences of brain injury, such as CTE (McKee et al., 2016; Lee et al., 2019; Schwab et al., 2021a). To date, only our lab has shown markers of DNA damage in post-mortem brains with a history of brain trauma, although it has been reported in AD as well (Wang et al., 2005; Lin et al., 2020). The brain bank used for our studies consists of male professional athletes with a history of brain trauma through involvement in contact sports such as hockey, American football, and boxing. Compared to healthy controls with no TBI history who showed no reactivity, TBI cases showed accumulation of γH2AX, a robust marker of DSBs, in ependymal cells lining the lateral ventricle, cortical and subcortical astrocytes, and oligodendrocytes of the subcortical white matter (Schwab et al., 2019a,b). In addition to evidence of DSBs in this cohort, gene expression analysis revealed significant downregulation of 37 genes involved in DNA repair including those involved in base-excision repair and homology-directed repair (Schwab et al., 2019b). The patients included in this cohort presented clinically with neurological symptoms ranging from the presence of a mood disorder to extreme behavioral changes and dementia, with some pathologically diagnosed with a neurodegenerative disease. However, it is also important to note that 50% of cases with no pathological diagnosis showed DNA damage in the form of DSBs, suggesting that DNA damage may precede neuropathological sequalae (Schwab et al., 2019b).

### DNA Damage Biomarkers in mTBI Patients

Many attempts have been made to identify robust biomarkers of brain trauma to be used for diagnosis, prognosis, or treatment-based decisions. Two studies have identified markers related to DNA damage in the blood of human patients with TBI, although whether they are derived from the brain, or the periphery remain unclear. In the blood plasma of patients with brain injury, a study in 2014 found that levels of cell free DNA correlated with severity of brain injury (mild vs. severe) (Shaked et al., 2014). Similarly, serum levels of 8-OHdG were found to correlate with mortality in patients with severe TBI, with patients who died from sustaining their injury having significantly elevated levels of this marker for oxidative DNA damage (Lorente et al., 2020).

### The Poly(ADP-Ribose) Polymerase (PARP) Response as an Example of DNA Damage in TBI

The poly(ADP-ribose) polymerase (PARP) enzyme is a DNA-binding protein which functions to detect DNA strand breaks induced by genotoxic agents. Several studies have identified PARP as a key player after brain trauma, further supporting the role of DNA damage in the pathophysiological response to injury. Indeed, mice with PARP knocked out are extremely sensitive to genomic instability caused by the alkylating agent N-methyl-N-nitrosourea or γ-irradiation (de Murcia et al., 1997). Indeed, PARP^−/−^ mice had significantly increased rates of mortality compared to PARP^+/+^ mice when treated with either genotoxic agent. These knockout mice also had higher rates of sister chromatid exchanges (SCEs) in bone marrow cells, indicating chromosomal instability in the absence of PARP (de Murcia et al., 1997). Primary fibroblasts lacking PARP *in vitro* fail to resume cell cycle progression and undergo apoptosis when treated with DNA damaging agents (de Murcia et al., 1997). The knockout mice and cells used in this study did not have altered phenotypes yet were sensitized to DNA damaging agents and so the authors concluded that PARP plays a central role in DNA repair, allowing survival signals to facilitate repair, and reduce genomic rearrangement and cell death processes (de Murcia et al., 1997). Following controlled cortical impact in rats, PARP is significantly increased in brain mitochondria (Lai et al., 2008), indicating the presence of mitochondrial DNA damage. Although PARP facilitates DNA repair, its persistent activation, as may be the case in TBI in which hypoxia and genotoxic stress can last for days following injury (Hill et al., 2017), can be detrimental. Indeed, PARP activation can lead to energy depletion and subsequent activation of cell death pathways, causing tissue damage (Andrabi et al., 2006). In this case, inhibiting PARP could be beneficial in preventing tissue damage and, indeed, this seems to be therapeutic in the context of TBI. In one study moderate controlled cortical impact injury was given to mice followed by treatment with either a PARP inhibitor or vehicle (Stoica et al., 2014). Inhibiting PARP after injury reduced neurodegeneration and lesion size and attenuated microglial activation compared to vehicle-treated injured mice. These changes were accompanied by improved motor function and cognitive abilities in the PARP-inhibitor group (Stoica et al., 2014). Similarly, another study using the fluid percussion injury model in rats showed that PARP inhibition results in reduced lesion size following moderate injury (LaPlaca et al., 2001). These studies on PARP further implicate DNA damage and excessive activation of DNA repair mechanisms as key players in the pathophysiological mechanism of TBI. As we know, persistent DNA damage and repair signals are known to cause stress-induced cellular senescence, for which there is a growing body of literature linking its involvement to TBI pathophysiology and the long-term repercussions of brain trauma.

## Cellular Senescence as a Pathophysiological Mechanism in mTBI

Several studies using experimental animal models of brain trauma have identified cellular senescence following injury. Tominaga et al. showed that in male mice who received moderate to severe injury using the controlled cortical impact model, SA-β-Gal positive cells were increased in the ipsilateral cerebrum at days 4, 7, and 14 post-injury, peaking at 7 days, compared to sham-treated mice (Tominaga et al., 2019). The authors also immunostained for several cell cycle markers including Cyclin D1, PCNA, p16, and p21, for which increased expressions are associated with DNA repair processes, cell cycle arrest, and regulation of cellular senescence (Tominaga et al., 2019). In injured mice, these cell cycle markers were significantly elevated in the ipsilateral cerebrum compared to shams up to 4 days post-injury and validated with mRNA expression analysis. p53, a potent inducer of cellular senescence, was significantly elevated in mRNA expression at 4, 7, and 14 days post-injury. The temporal pattern suggests that following TBI, the cell cycle is initially activated by DNA repair signals, followed by activation of cellular senescence. Thus, this study has identified two senescence pathways as being activated after TBI in the ipsilateral cerebrum (adjacent to the injury site): the p16- and p53-mediated pathways. The authors suggest that these pathways may be active in astrocytes and neurons, respectively, and further research using techniques like single-cell sequencing will be helpful in elucidating this question (Tominaga et al., 2019).

In a different study, Ritzel et al. used the same model as above in mice to compare markers of cellular senescence in microglia between young (3 month old) and aged (12 month old) mice (Ritzel et al., 2019). The authors showed that moderate TBI results in elevated gene and protein expression levels of senescence markers in microglia, such as B-cell lymphoma 2 (BCL-2), p16, p21, lipofuscin, and γH2AX 72 h post-injury in both the young and old mice, with old mice showing significantly higher expression of BCL-2, p16, and γH2AX. This study therefore has not only similarly identified the p16-RB senescence pathway as being activated in microglia following moderate TBI but has also provided insight into the effects of aging on TBI (Ritzel et al., 2019). Indeed, as we know, senescent cells accumulate in the brain with age, including microglia, and are linked to age-related neuroinflammation. This baseline level of cellular senescence in the aged brain and additional burden from TBI-induced senescence could therefore explain the functional consequences of TBI in the aged brain, including increased rates of mortality and poor clinical outcomes reported in older patients (Thompson et al., 2006).

In a blast exposure model using adult male rats, Arun et al. exposed animals to a single or repeated (two) moderate TBI and assessed cellular senescence markers at 24 h, 1 month, or 1 year post-injury (Arun et al., 2020). Injured mice showed significantly increased activity of SA-β-Gal at 24 h and 1 month post-injury in regions including the motor cortex, auditory cortex, dorsolateral thalamus, superior colliculus, geniculate nucleus, ventral thalamic nucleus, and hippocampus compared to sham controls. No differences between the single or repeated injury groups were identified. In addition, the authors found significantly decreased mRNA expression of senescence marker protein-30 (SMP-30) and significantly elevated expression of p21 in the cortex at 1 month post-injury. Again, this study supports previous studies showing p21-mediated cellular senescence as an activated pathway following models of TBI (Arun et al., 2020).

In mice, our lab has supported these findings to show evidence of cellular senescence even after mild injury comparable to a concussion (Schwab et al., 2021b). Using a closed skull impact model, mice were exposed to repeated (one injury daily for 3 consecutive days) mTBI and examined for markers of cellular senescence and DNA damage at 24 h and 7 days post-injury. At 24 h, injured mice showed gene expression profiling consistent with activation of cell cycle markers and the DNA damage response to double-strand DNA breaks, single-strand breaks, and oxidative lesions. By 7 days, the gene expression profile of injured mice was consistent with cellular senescence, including elevation of the BCL-2 pro-senescence signaling pathway and reduced DNA repair factors. By 7 days, injured mice also showed elevated mRNA expression of interleukin 1β (IL1β), interleukin 10 (IL10), p21, p53-binding protein 1 (53BP1), and elevated protein expression of p53 in the ipsilateral cortex. These changes suggest that even in a repeated mild injury, DNA damage-induced cellular senescence is an aftermath of TBI, particularly the p21-mediated pathway (Schwab et al., 2021b).

Lastly, our lab has previously shown evidence of cellular senescence in post-mortem human brains with a history of brain trauma (Schwab et al., 2019b), indicating that the experimental models outlined above are clinically relevant. In this study, 38 cases with mTBI history and 10 healthy controls were assessed for clinical presentation, neuropathological changes, load of DNA damage (γH2AX), and various markers of cellular senescence. In this cohort, 28/38 (74%) had γH2AX reactivity in ependymal cells, astrocytes, and/or oligodendrocytes throughout the brain, while no reactivity was seen in control brains. Using immunohistochemistry, key features of senescence were identified in brains with DNA damage, cell body swelling and beading, and loss of H3K27Me3 (trimethylation at lysine 27 of histone h3) and Lamin B1 expression. Gene expression analysis revealed reduced expression of DNA repair proteins in cases compared to controls, as well as significantly elevated expression of pro-inflammatory SASP factors such as IL1β, interleukin 6 (IL6), CXCL1 (chemokine ligand 1), and CCL8 (chemokine ligand 8) among others. This study showed that human brains with a history of trauma have evidence of cellular senescence in glial cells, even in some cases with no neuropathological diagnosis despite having clinical symptoms and neurological dysfunction during life (Schwab et al., 2019b). Cellular senescence could therefore be the driver of clinical symptoms and long-term neurological problems after trauma, which will be discussed subsequently. Additionally, as this study identified reduced DNA repair pathways in the injured brain, this study suggests that inefficient DNA repair may further confer susceptibility to brain dysfunction after TBI. This poses important questions for understanding risk factors, as many DNA repair factors are naturally polymorphic or may be affected by factors such as sex (Fischer and Riddle, 2018; Leung and Hazrati, 2021), age (Fischer and Riddle, 2018), and lifestyle factors such as substance use (Madden et al., 1979).

Most of the above-mentioned studies are critical in our understanding of cellular senescence after brain injury and have provided enormous support for its involvement in the pathophysiological consequences of TBI. However, some limitations do exist including the lack of studies which include female animals, and the identification of cell-type specific mechanisms, which could be identified using advanced techniques such as single-cell sequencing.

## Molecular and Cellular Changes After mTBI: Substrates of Senescence?

**Figure 1:**
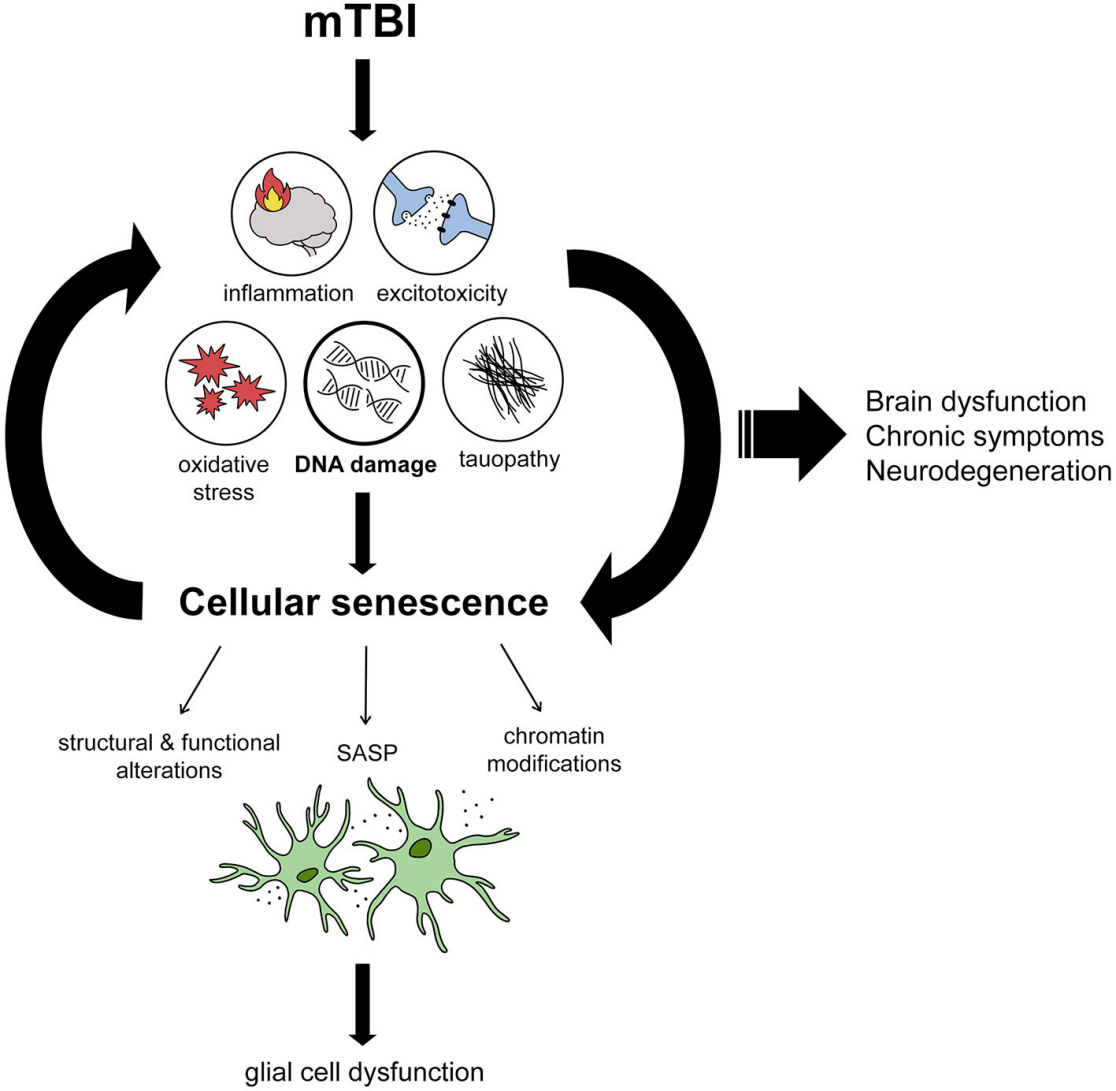
Proposed mechanism by which mTBI leads to brain dysfunction through DNA damage-induced cellular senescence. From reviewing the literature, we propose several immediate effects of mTBI including inflammation, excitotoxicity, oxidative stress, tau dysfunction, and DNA damage which encompasses all the aforementioned features. DNA damage induces cellular senescence when persistent, leading to structural and functional alterations in cells, chromatin modifications changing gene expression signatures, and inflammation by way of the senescence-associated secretory phenotype (SASP). We propose that senescence mostly affects glial cells, resulting in their dysfunction. Cellular senescence acts as a positive feedback loop, with reduced DNA repair and SASP leading to chronic levels of DNA damage and inflammation. We propose that the induction of cellular senescence in this way leads to chronic brain dysfunction and symptoms that are associated with mTBI, including neurodegenerative diseases in the long-term.

While the upstream mechanisms causing dysfunction after mTBI are still being explored, several molecular repercussions of mTBI are well-studied and characterized. Like the clinical manifestations of mTBI discussed earlier, these molecular changes may be substrates of cellular senescence (Figure 1). One of the most immediate and well-studied repercussions after mTBI is oxidative stress, otherwise known as a state of metabolism in which the production of reactive oxygen species (ROS) substantially offsets the production of antioxidant defenses (Higgins et al., 2010). The effect of oxidative stress on the brain is detrimental, as this leads to the accumulation of oxidative damage, SSBs, and DSBs to DNA [99], as well as impairment of DNA repair machinery (Wu et al., 2017). The accumulation of DNA damage in human brains and animal models with brain trauma may therefore be initiated by the oxidative stress process. Neuroinflammation is another well-studied repercussion of mTBI for which cellular senescence may be the upstream and downstream mechanism. Indeed, several studies have identified chronic low-level inflammation in both human brains (Smith et al., 2013) and animal models (Chiu et al., 2016) with brain trauma. Several of the factors implicated in mTBI-associated neuroinflammation are also considered to be SASP factors, such as IL1β, IL6, TNFα, and matrix metallopeptidase 12 (MMP-12), among others (Woodcock and Morganti-Kossmann, 2013). Studies on mTBI have also repeatedly shown evidence of glutamate excitotoxicity in neurons after injury (Luo et al., 2019; Tehse and Taghibiglou, 2019), and this mechanism has been suggested as the cause of mTBI-related seizures in a subset of patients (Pitkänen and Immonen, 2014). Importantly, senescent astrocytes have been shown to cause glutamate excitotoxicity and subsequent neuronal death *in vitro* via the downregulation of genes encoding glutamate transporters on senescent astrocytes (Limbad et al., 2020). In terms of the long-term consequences of mTBI, such as increased risk of various tauopathies, cellular senescence has been proposed as a driver of p-tau aggregation (Musi et al., 2018). Indeed, neurofibrillary tangle (NFT)-containing neurons from post-mortem AD brains have a senescence-consistent expression profile, and in AD transgenic mouse models treatment with senolytic drugs to remove senescent cells resulted in reduced NFT density and reduced neurodegeneration (Mendelsohn and Larrick, 2018; Musi et al., 2018). Similarly, in the MAPTP^301S^PS19 mouse model of tau-dependent neurodegeneration, Bussian et al. showed that elimination of senescent cells prevents hyperphosphorylation and accumulation of tau, while improving cognitive impairment (Bussian et al., 2018). This study indicates that senescent cells may play a causal role in p-tau accumulation and aggregation and is a potential treatment target for these pathologies.

Cellular senescence has also been associated with amyloid pathology, the accumulation of the neurotoxic oligomer characteristic of Alzheimer's disease, but which is also present in brains with CTE (Stein et al., 2014). Indeed, amyloid β was shown to accelerate cellular senescence, marked by increased expression of p16, in a 5XFAD mouse model of Alzheimer's (Wei et al., 2016). Similarly, brains of both AD patients and an AD mouse model showed marked evidence of cellular senescence specifically in oligodendrocyte precursor cells (Zhang et al., 2019). This study suggests that amyloid β aggregation promotes cellular senescence and found that the use of senolytic treatment to eliminate senescent cells results in reduced neuroinflammation, Aβ burden, and cognitive deficits in the mouse model (Zhang et al., 2019). In fact, cellular senescence has recently been proposed as an essential component of the amyloid cascade hypothesis, with elimination of senescent cells suggested as a possible treatment rather than the use of Aβ-targeting antibodies (Walton et al., 2020). While the precise relationship between Aβ and cellular senescence is currently being explored, the evidence strongly suggests a synergistic relationship with both components positively feeding back to each other. Thus, targeting the early initiators of cellular senescence, namely DNA damage in the case of mTBI, or cellular senescence in its early phases (prior to the accumulation of neurotoxic protein aggregates) may be the optimal approach for such a disease state.

Collectively, these studies show that many of the repercussions of cellular senescence, including DNA damage, neuroinflammation, excitotoxicity, and tauopathy, are also evident following mTBI. Therefore, the emerging research on the effects of cellular senescence in the brain may indicate this process as an upstream mechanism causing many of the molecular and cellular changes associated with mTBI.

## Conclusion

There is a growing body of evidence showing DNA damage and cellular senescence in various forms of TBI, including mTBI. As both the clinical and molecular substrates of mTBI are associated with cellular senescence, this evidence suggests that cellular senescence may be an upstream overriding mechanism at play in mTBI and may represent a therapeutic target for early interventions. Furthermore, individual and sex differences in DNA repair capacities may help explain heterogeneity in the response to TBI, particularly mTBI and concussions. We emphasize that the potential therapies mentioned in this review are for early intervention, with the goal of preventing long-term consequences of brain injury rather than strategies to reverse neuropathological changes induced by head trauma. This limits the use of DNA damage/senescence-targeting strategies to patients in early periods (i.e., directly following head impact) rather than patients with remote, chronic head trauma histories. Therapies designed for reversal of neuropathological changes (such as tau and amyloid) associated with head trauma are yet to be elucidated and are beyond the scope of this review. However, with that said, senolytic therapies may still be clinically beneficial to chronic patients with evidence of pathological entities, as this review has summarized the detrimental effects of senescence even in the presence of neurodegenerative diseases. Indeed the chronic effects inflicted by senescent cells may be the basis of functional deficits, and therefore targeting senescence at any stage may be helpful.

## Author Contributions

All authors took part in literature search, manuscript preparation, and editing.

## Conflict of Interest

The authors declare that the research was conducted in the absence of any commercial or financial relationships that could be construed as a potential conflict of interest.

## Publisher's Note

All claims expressed in this article are solely those of the authors and do not necessarily represent those of their affiliated organizations, or those of the publisher, the editors and the reviewers. Any product that may be evaluated in this article, or claim that may be made by its manufacturer, is not guaranteed or endorsed by the publisher.

## Abbreviations

8-OHdG: 8-hydroxy-2′-deoxyguanosine
AD: Alzheimer's Disease
ATM: ataxia telangiectasia mutated
BCL-2: B-cell lymphoma 2
CCI: controlled cortical impact
CTE: chronic traumatic encephalopathy
DDR: DNA damage response
DSB: double strand break
GCS: Glasgow Comma Scale
GSEA: gene set enrichment analysis
H2AX: histone family member X
IL1β: interleukin 1β
IL6: interleukin 6
IL10: interleukin 10
LOC: loss of consciousness
mTBI: mild traumatic brain injury
NFT: neurofibrillary tangle
PANT: DNA polymerase-I-mediated biotin-dATP nick-translation
PARP: poly(ADP-ribose) polymerase
PCS: post-concussive syndrome
PD: Parkinson's Disease
ROS: reactive oxygen species
SASP: senescence-associated secretory phenotype
SA-β-Gal: senescence-associated β-galactosidase
SCE: sister chromatid exchange
SSB: single strand break
TBI: traumatic brain injury
TUNEL: terminal deoxynucleotide transferase-mediated dUTP nick-end labeling.

## References

[B1] Aird K. M. Zhang R. (2013). Detection of senescence-associated heterochromatin foci (SAHF). Methods Mol. Biol. 965, 185–196. 10.1007/978-1-62703-239-1_12 23296659 PMC3552318

[B2] Andrabi S. A. Kim N. S. Yu S. W. Wang H. Koh D. W. Sasaki M.. (2006). Poly(ADP-ribose) (PAR) polymer is a death signal. Proc. Natl. Acad. Sci. U. S. A. 103, 18308–18313. 10.1073/pnas.0606526103 17116882 PMC1838747

[B3] Arun P. Rossetti F. Wilder D. M. Sajja S. Van Albert S. A. Wang Y.. (2020). Blast exposure leads to accelerated cellular senescence in the rat brain. Front. Neurol. 11:438. 10.3389/fneur.2020.00438 32508743 PMC7253679

[B4] Baker D. J. Petersen R. C. (2018). Cellular senescence in brain aging and neurodegenerative diseases: evidence and perspectives. J. Clin. Invest. 128, 1208–1216. 10.1172/JCI95145 29457783 PMC5873891

[B5] Barnes D. E. Byers A. L. Gardner R. C. Seal K. H. Boscardin W. J. Yaffe K. (2018). Association of mild traumatic brain injury with and without loss of consciousness with dementia in US military veterans. JAMA Neurol. 75, 1055–1061. 10.1001/jamaneurol.2018.0815 29801145 PMC6143113

[B6] Blyth B. J. Bazarian J. J. (2010). Traumatic alterations in consciousness: traumatic brain injury. Emerg. Med. Clin. North Am. 28, 571–594. 10.1016/j.emc.2010.03.003 20709244 PMC2923650

[B7] Borgesius N. Z. de Waard M. C. van der Pluijm I. Omrani A. Zondag G. C. van der Horst G. T.. (2011). Accelerated age-related cognitive decline and neurodegeneration, caused by deficient DNA repair. J. Neurosci. 31,12543–12553. 10.1523/JNEUROSCI.1589-11.2011 21880916 PMC6703271

[B8] Burma S. Chen B. P. Murphy M. Kurimasa A. Chen D. J. (2001). ATM phosphorylates histone H2AX in response to DNA double-strand breaks. J. Biol. Chem. 276, 42462–42467. 10.1074/jbc.C100466200 11571274

[B9] Bussian T. J. Aziz A. Meyer C. F. Swenson B. L. van Deursen J. M. Baker D. J. (2018). Clearance of senescent glial cells prevents tau-dependent pathology and cognitive decline. Nature 562, 578–582. 10.1038/s41586-018-0543-y 30232451 PMC6206507

[B10] Cadet J. Davies K. (2017). Oxidative DNA damage and repair: an introduction. Free Rad. Biol. Med. 107, 2–12 10.1016/j.freeradbiomed.2017.03.030 28363603 PMC5510741

[B11] Carrol L. J. Cassidy J. D. Holm L. Kraus J. Coronado V. G. WHO Collaborating Centre Task Force on Mild Traumatic Brain Injury (2004). Methodological issues and research recommendations for mild traumatic brain injury: the WHO Collaborating Centre Task Force on Mild Traumatic Brain Injury. J. Rehabil. Med. 43, 113–125. 10.1080/16501960410023877 15083875

[B12] Carroll J. E. Cole S. W. Seeman T. E. Breen E. C. Witarama T. Arevalo J.. (2016). Partial sleep deprivation activates the DNA damage response (DDR) and the senescence-associated secretory phenotype (SASP) in aged adult humans. Brain Behav. Immun. 51, 223–229. 10.1016/j.bbi.2015.08.024 26336034 PMC4679552

[B13] Chinta S. J. Lieu C. A. Demaria M. Laberge R. M. Campisi J. Andersen J. K. (2013). Environmental stress, ageing and glial cell senescence: a novel mechanistic link to Parkinson's disease?. J. Intern. Med. 273, 429–436. 10.1111/joim.12029 23600398 PMC3633085

[B14] Chiu C. C. Liao Y. E. Yang L. Y. Wang J. Y. Tweedie D. Karnati. (2016). Neuroinflammation in animal models of traumatic brain injury. J. Neurosci. Methods 272, 38–49. 10.1016/j.jneumeth.2016.06.018 27382003 PMC5201203

[B15] Clark R. S. B. Chen M. Kochanek P. M. Watkins S. C. Jin K. L. Draviam R.. (2001). Detection of single- and double-strand DNA breaks after traumatic brain injury in rats: comparison of *in situ* labeling techniques using DNA polymerase I, the Klenow fragment of DNA polymerase I, and terminal deoxynucleotidyl transferase. J. Neurotrauma 18, 675–689. 10.1089/089771501750357627 11497094

[B16] Cohen J. Torres C. (2019). Astrocyte senescence: evidence and significance. Aging Cell 18:e12937. 10.1111/acel.12937 30815970 PMC6516680

[B17] Collin G. Huna A. Warnier M. Flaman J. M. Bernard D. (2018). Transcriptional repression of DNA repair genes is a hallmark and a cause of cellular senescence. Cell Death Dis. 9:259. 10.1038/s41419-018-0300-z 29449545 PMC5833687

[B18] Conde J. R. Streit W. J. (2006). Effect of aging on the microglial response to peripheral nerve injury. Neurobiol. Aging 27, 1451–1461 10.1016/j.neurobiolaging.2005.07.012 16159684

[B19] de Murcia J. M. Niedergang C. Trucco C. Ricoul M. Dutrillaux B. Mark M.. (1997). Requirement of poly(ADP-ribose) polymerase in recovery from DNA damage in mice and in cells. Proc. Natl. Acad. Sci. U. S. A. 94, 7303–7307. 10.1073/pnas.94.14.7303 9207086 PMC23816

[B20] Deutsch M. B. Mendez M. F. Teng E. (2015). Interactions between traumatic brain injury and frontotemporal degeneration. Dement. Geriatr. Cogn. Disord. 39, 143–153. 10.1159/000369787 25531628 PMC4427348

[B21] Di Micco R. Krizhanovsky V. Baker D. d'Adda di Fagagna F. (2021). Cellular senescence in ageing: from mechanisms to therapeutic opportunities. Nat. Rev. Mol. Cell Biol. 22, 75–95. 10.1038/s41580-020-00314-w 33328614 PMC8344376

[B22] Dimri G. P. Lee X. Basile G. Acosta M. Scott G. Roskelley C.. (1995). A biomarker that identifies senescent human cells in culture and in aging skin *in vivo*. Proc. Natl. Acad. Sci. U. S. A. 92, 9363–9367. 10.1073/pnas.92.20.9363 7568133 PMC40985

[B23] Diniz B. S. Reynolds C. F III Sibille E. Lin C. W. Tseng G. Lotrich F.. (2017). Enhanced molecular aging in late-life depression: the senescent-associated secretory phenotype. Am. J. Geriatr. Psychiatry 25, 64–72. 10.1016/j.jagp.2016.08.018 27856124 PMC5164865

[B24] Dou Z. Ghosh K. Vizioli M. G. Zhu J. Sen P. Wangensteen K. J.. (2017). Cytoplasmic chromatin triggers inflammation in senescence and cancer. Nature 550, 402–406. 10.1038/nature24050 28976970 PMC5850938

[B25] Escartin C. Galea E. Lakatos A. O'Callaghan J. P. Petzold G. C. Serrano-Pozo A.. (2021). Reactive astrocyte nomenclature, definitions, and future directions. Nat. Neurosci. 24, 312–325. 10.1038/s41593-020-00783-4 33589835 PMC8007081

[B26] Fielder E. von Zglinicki T. Jurk D. (2017). The DNA damage response in neurons: die by apoptosis or survive in a senescence-like state?. J. Alzheimer Dis. 60, S107–S131. 10.3233/JAD-161221 28436392

[B27] Fischer K. E. Riddle N. C. (2018). Sex differences in aging: genomic instability. J. Gerontol. A Biol. Sci. Med. Sci. 73, 166–174. 10.1093/gerona/glx105 28575157 PMC5861920

[B28] Freund A. Laberge R. M. Demaria M. Campisi J. (2012). Lamin B1 loss is a senescence-associated biomarker. Mol. Biol. Cell 23, 2066–2075. 10.1091/mbc.e11-10-0884 22496421 PMC3364172

[B29] Freund A. Orjalo A. V. Desprez P. Y. Campisi J. (2010). Inflammatory networks during cellular senescence: causes and consequences. Trends Mol. Med. 16, 238–246. 10.1016/j.molmed.2010.03.003 20444648 PMC2879478

[B30] Fujimura M. Morita-Fujimura Y. Noshita N. Yoshimoto T. Chan P. H. (2000). Reduction of the DNA base excision repair protein, XRCC1, may contribute to DNA fragmentation after cold injury-induced brain trauma in mice. Brain Res. 869, 105–111. 10.1016/S0006-8993(00)02375-1 10865064

[B31] Gardner R. C. Burke J. F. Nettiksimmons J. Goldman S. Tanner C. M. Yaffe K. (2015). Traumatic brain injury in later life increases risk for Parkinson disease. Ann. Neurol. 77, 987–995. 10.1002/ana.24396 25726936 PMC4447556

[B32] Garinis G. A. van der Horst G. T. Vijg J. Hoeijmakers J. H. (2008). DNA damage and ageing: new-age ideas for an age-old problem. Nat. Cell Biol. 10, 1241–1247. 10.1038/ncb1108-1241 18978832 PMC4351702

[B33] Hall E. D. Sullivan P. G. Gibson T. R. Pavel K. M. Thompson B. M. Scheff S. W. (2005). Spatial and temporal characteristics of neurodegeneration after controlled cortical impact in mice: more than a focal brain injury. J. Neurotrauma 22, 252–265. 10.1089/neu.2005.22.252 15716631

[B34] Han X. Zhang T. Liu H. Mi Y. Gou X. (2020). Astrocyte senescence and Alzheimer's disease: a review. Front. Aging Neurosci. 12:148. 10.3389/fnagi.2020.00148 32581763 PMC7297132

[B35] Higgins G. C. Beart P. M. Shin Y. S. Chen M. J. Cheung N. S. Nagley P. (2010). Oxidative stress: emerging mitochondrial and cellular themes and variations in neuronal injury. J. Alzheimer Dis. 20, S453–S473. 10.3233/JAD-2010-100321 20463398

[B36] Hill R. L. Singh I. N. Wang J. A. Hall E. D. (2017). Time courses of post-injury mitochondrial oxidative damage and respiratory dysfunction and neuronal cytoskeletal degradation in a rat model of focal traumatic brain injury. Neurochem. Int. 111, 45–56. 10.1016/j.neuint.2017.03.015 28342966 PMC5610595

[B37] Hiploylee C. Dufort P. A. Davis H. S. Wennberg R. A. Tartaglia M. C. Mikulis D.. (2017). Longitudinal study of postconcussion syndrome: not everyone recovers. J. Neurotrauma 34, 1511–1523. 10.1089/neu.2016.4677 27784191 PMC5397249

[B38] Hyder A. A. Wunderlich C. A. Puvanachandra P. Gururaj G. Kobusingye O. C. (2007). The impact of traumatic brain injuries: a global perspective. NeuroRehabilitation 22, 341–353. 10.3233/NRE-2007-22502 18162698

[B39] Iacono D. Raiciulescu S. Olsen C. Perl D. P. (2021). Traumatic brain injury exposure lowers age of cognitive decline in AD and non-AD conditions. Front. Neurol. 12:573401. 10.3389/fneur.2021.573401 34054681 PMC8153372

[B40] Ito T. Teo Y. V. Evans S. A. Neretti N. Sedivy J. M. (2018). Regulation of cellular senescence by polycomb chromatin modifiers through distinct DNA damage- and histone methylation-dependent pathways. Cell Rep. 22, 3480–3492. 10.1016/j.celrep.2018.03.002 29590617 PMC5915310

[B41] Jafari S. Etminan M. Aminzadeh F. Samii A. (2013). Head injury and risk of Parkinson disease: a systematic review and meta-analysis. Mov. Disord. 28,1222–1229. 10.1002/mds.25458 23609436

[B42] Koellhoffer E. C. McCullough L. D. Ritzel R. M. (2017). Old maids: aging and its impact on microglia function. Int. J. Mol. Sci. 18:769. 10.3390/ijms18040769 28379162 PMC5412353

[B43] Lai Y. Chen Y. Watkins S. C. Nathaniel P. D. Guo F. Kochanek P. M.. (2008). Identification of poly-ADP-ribosylated mitochondrial proteins after traumatic brain injury. J. Neurochem. 104, 1700–1711. 10.1111/j.1471-4159.2007.05114.x 17996029

[B44] LaPlaca M. C. Zhang J. Raghupathi R. Li J. H. Smith F. Bareyre F. M.. (2001). Pharmacologic inhibition of poly(ADP-ribose) polymerase is neuroprotective following traumatic brain injury in rats. J. Neurotrauma 18, 369–376. 10.1089/089771501750170912 11336438

[B45] Lee B. Newberg A. (2005). Neuroimaging in traumatic brain imaging. NeuroRx 2, 372–383. 10.1602/neurorx.2.2.372 15897957 PMC1064998

[B46] Lee E. B. Kinch K. Johnson V. E. Trojanowski J. Q. Smith D. H. Stewart W. (2019). Chronic traumatic encephalopathy is a common co-morbidity, but less frequent primary dementia in former soccer and rugby players. Acta Neuropathol. 138, 389–399. 10.1007/s00401-019-02030-y 31152201 PMC6689293

[B47] Leung E. Hazrati L. N. (2021). Breast cancer type 1 and neurodegeneration: consequences of deficient DNA repair. Brain Commun. 3:fcab117. 10.1093/braincomms/fcab117 34222870 PMC8242133

[B48] Li Z. Pearlman A. H. Hsieh P. (2016). DNA mismatch repair and the DNA damage response. DNA Repair. 38, 94–101. 10.1016/j.dnarep.2015.11.019 26704428 PMC4740233

[B49] Limbad C. Oron T. R. Alimirah F. Davalos A. R. Tracy T. E. Gan L.. (2020). Astrocyte senescence promotes glutamate toxicity in cortical neurons. PLoS ONE 15:e0227887. 10.1371/journal.pone.0227887 31945125 PMC6964973

[B50] Lin X. Kapoor A. Gu Y. Chow M. J. Peng J. Zhao K.. (2020). Contributions of DNA damage to Alzheimer's disease. Int. J. Mol. Sci. 21:1666. 10.3390/ijms21051666 32121304 PMC7084447

[B51] Lorente L. Martín M. M. González-Rivero A. F. Pérez-Cejas A. Abreu-González P. Ramos L.. (2020). Association between DNA and RNA oxidative damage and mortality of patients with traumatic brain injury. Neurocrit. Care 32, 790–795. 10.1007/s12028-019-00800-w 31385181

[B52] Luo P. Li X. Wu X. Dai S. Yang Y. Xu H.. (2019). Preso regulates NMDA receptor-mediated excitotoxicity via modulating nitric oxide and calcium responses after traumatic brain injury. Cell Death Dis. 10:496. 10.1038/s41419-019-1731-x 31235685 PMC6591282

[B53] Madabhushi R. Pan L. Tsai L. H. (2014). DNA damage and its links to neurodegeneration. Neuron 83, 266–832. 10.1016/j.neuron.2014.06.034 25033177 PMC5564444

[B54] Madden J. J. Falek A. Shafer D. A. Glick J. H. (1979). Effects of opiates and demographic factors on DNA repair synthesis in human leukocytes. Proc. Natl. Acad. Sci. U. S. A. 76, 5769–5773. 10.1073/pnas.76.11.5769 293681 PMC411732

[B55] Maroon J. C. Winkelman R. Bost J. Amos A. Mathyssek C. Miele V. (2015). Chronic traumatic encephalopathy in contact sports: a systematic review of all reported pathological cases. PLoS ONE 10:e0117338. 10.1371/journal.pone.0117338 25671598 PMC4324991

[B56] Martínez-Cué C. Rueda N. (2020). Cellular senescence in neurodegenerative diseases. Front. Cell. Neurosci. 14:16. 10.3389/fncel.2020.00016 32116562 PMC7026683

[B57] Max J. E. Keatley E. Wilde E. A. Bigler E. D. Schachar R. J. Saunders A. E.. (2012). Depression in children and adolescents in the first 6 months after traumatic brain injury. Int. J. Dev. Neurosci. 30, 239–245. 10.1016/j.ijdevneu.2011.12.005 22197971 PMC3322312

[B58] McInnes K. Friesen C. L. MacKenzie D. E. Westwood D. A. Boe S. G. (2017). Mild Traumatic Brain Injury (mTBI) and chronic cognitive impairment: a scoping review. PLoS ONE 12:e0174847. 10.1371/journal.pone.0174847 28399158 PMC5388340

[B59] McKee A. C. Cairns N. J. Dickson D. W. Folkerth R. D. Keene C. D. Litvan I.. (2016). The first NINDS/NIBIB consensus meeting to define neuropathological criteria for the diagnosis of chronic traumatic encephalopathy. Acta Neuropathol. 131, 75–86. 10.1007/s00401-015-1515-z 26667418 PMC4698281

[B60] Mendelsohn A. R. Larrick J. W. (2018). Cellular senescence as the key intermediate in tau-mediated neurodegeneration. Rejuvenation Res. 21, 572–579. 10.1089/rej.2018.2155 30489222

[B61] Mendez D. R. Cherian L. Moore N. Arora T. Liu P. K. Robertson C. S. (2004). Oxidative DNA lesions in a rodent model of traumatic brain injury. J. Trauma 56, 1235–1240. 10.1097/01.TA.0000130759.62286.0E 15211131

[B62] Mez J. Solomon T. M. Daneshvar D. H. Murphy L. Kiernan P. T. Montenigro P. H.. (2015). Assessing clinicopathological correlation in chronic traumatic encephalopathy: rationale and methods for the UNITE study. Alzheimer Res. Therapy 7:62. 10.1186/s13195-015-0148-8 26455775 PMC4601147

[B63] Moreno-Blas D. Gorostieta-Salas E. Pommer-Alba A. Muciño-Hernández G. Gerónimo-Olvera C. Maciel-Barón L. A.. (2019). Cortical neurons develop a senescence-like phenotype promoted by dysfunctional autophagy. Aging 11, 6175–6198. 10.18632/aging.102181 31469660 PMC6738425

[B64] Morita-Fujimura Y. Fujimura M. Kawase M. Chan P. H. (1999). Early decrease in apurinic/apyrimidinic endonuclease is followed by DNA fragmentation after cold injury-induced brain trauma in mice. Neuroscience 93, 1465–1473. 10.1016/S0306-4522(99)00231-6 10501471

[B65] Mortimer J. A. van Duijn C. M. Chandra V. Fratiglioni L. Graves A. B. Heyman A.. (1991). Head trauma as a risk factor for Alzheimer's disease: a collaborative re-analysis of case-control studies. Int. J. Epidemiol. 20, S28–35. 10.1093/ije/20.Supplement_2.S28 1833351

[B66] Musi N. Valentine J. M. Sickora K. R. Baeuerle E. Thompson C. S. Shen Q.. (2018). Tau protein aggregation is associated with cellular senescence in the brain. Aging Cell 17:e12840. 10.1111/acel.12840 30126037 PMC6260915

[B67] Ogrodnik M. Zhu Y. Langhi L. Tchkonia T. Krüger P. Fielder E.. (2019). Obesity-induced cellular senescence drives anxiety and impairs neurogenesis. Cell Metabo. 29, 1061–1077. 10.1016/j.cmet.2018.12.008 30612898 PMC6509403

[B68] Ojo J. O. Mouzon B. Greenberg M. B. Bachmeier C. Mullan M. Crawford F. (2013). Repetitive mild traumatic brain injury augments tau pathology and glial activation in aged hTau mice. J. Neuropathol. Exp. Neurol. 72, 137–151. 10.1097/NEN.0b013e3182814cdf 23334597

[B69] Pertusa M. García-Matas S. Rodríguez-Farré E. Sanfeliu C. Cristòfol R. (2007). Astrocytes aged *in vitro* show a decreased neuroprotective capacity. J. Neurochem. 101, 794–805. 10.1111/j.1471-4159.2006.04369.x 17250685

[B70] Pitkänen A. Immonen R. (2014). Epilepsy related to traumatic brain injury. Neurotherapeutics 11, 286–296. 10.1007/s13311-014-0260-7 24554454 PMC3996118

[B71] Rayess H. Wang M. B. Srivatsan E. S. (2012). Cellular senescence and tumor suppressor gene p16. Int. J. Cancer 130, 1715–1725. 10.1002/ijc.27316 22025288 PMC3288293

[B72] Ritzel R. M. Doran S. J. Glaser E. P. Meadows V. E. Faden A. I. Stoica B. A.. (2019). Old age increases microglial senescence, exacerbates secondary neuroinflammation, and worsens neurological outcomes after acute traumatic brain injury in mice. Neurobiol. Aging 77, 194–206. 10.1016/j.neurobiolaging.2019.02.010 30904769 PMC6486858

[B73] Rufini A. Tucci P. Celardo I. Melino G. (2013). Senescence and aging: the critical roles of p53. Oncogene 32, 5129–5143. 10.1038/onc.2012.640 23416979

[B74] Ryu W. H. Feinstein A. Colantonio A. Streiner D. L. Dawson D. R. (2009). Early identification and incidence of mild TBI in Ontario. Can. J. Neurol. Sci. 36, 429–435. 10.1017/S0317167100007745 19650352

[B75] Schwab N. Grenier K. Hazrati L. N. (2019b). DNA repair deficiency and senescence in concussed professional athletes involved in contact sports. Acta Neuropathol. Commun. 7:182. 10.1186/s40478-019-0822-3 31727161 PMC6857343

[B76] Schwab N. Ju Y. Hazrati L. N. (2021b). Early onset senescence and cognitive impairment in a murine model of repeated mTBI. Acta Neuropathol. Commun. 9:82. 10.1186/s40478-021-01190-x 33964983 PMC8106230

[B77] Schwab N. Tator C. Hazrati L. N. (2019a). DNA damage as a marker of brain damage in individuals with history of concussions. Lab. Invest. 99, 1008–1018. 10.1038/s41374-019-0199-8 30760862

[B78] Schwab N. Wennberg R. Grenier K. Tartaglia C. Tator C. Hazrati L. N. (2021a). Association of position played and career duration and chronic traumatic encephalopathy at autopsy in elite football and hockey players. Neurology 96, e1835–e1843. 10.1212/WNL.0000000000011668 33627496 PMC8105967

[B79] Setnik L. Bazarian J. J. (2007). The characteristics of patients who do not seek medical treatment for traumatic brain injury. Brain injury 21, 1–9. 10.1080/02699050601111419 17364514

[B80] Shaked G. Douvdevani A. Yair S. Zlotnik A. Czeiger D. (2014). The role of cell-free DNA measured by a fluorescent test in the management of isolated traumatic head injuries. Scand. J. Trauma Resusc. Emerg. Med. 22:21. 10.1186/1757-7241-22-21 24641833 PMC4000614

[B81] Simpson J. E. Ince P. G. Matthews F. E. Shaw P. J. Heath P. R. Brayne C.. (2015). A neuronal DNA damage response is detected at the earliest stages of Alzheimer's neuropathology and correlates with cognitive impairment in the medical research council's cognitive function and ageing study ageing brain cohort. Neuropathol. Appl. Neurobiol. 41, 483–496. 10.1111/nan.12202 25443110 PMC4861215

[B82] Smith C. Gentleman S. M. Leclercq P. D. Murray L. S. Griffin W. S. Graham D. I.. (2013). The neuroinflammatory response in humans after traumatic brain injury. Neuropathol. Appl. Neurobiol. 39, 654–666. 10.1111/nan.12008 23231074 PMC3833642

[B83] Soto-Gamez A. Quax W. J. Demaria M. (2019). Regulation of survival networks in senescent cells: from mechanisms to interventions. J. Mol. Biol. 431, 2629–2643. 10.1016/j.jmb.2019.05.036 31153901

[B84] Stein T. D. Alvarez V. E. McKee A. C. (2014). Chronic traumatic encephalopathy: a spectrum of neuropathological changes following repetitive brain trauma in athletes and military personnel. Alzheimers. Res. Ther. 6, 1–11. 10.1186/alzrt234 24423082 PMC3979082

[B85] Stoica B. A. Loane D. J. Zhao Z. Kabadi S. V. Hanscom M. Byrnes K. R.. (2014). PARP-1 inhibition attenuates neuronal loss, microglia activation and neurological deficits after traumatic brain injury. J. Neurotrauma 31, 758–772. 10.1089/neu.2013.3194 24476502 PMC3967421

[B86] Stolz A. Ertych N. Bastians H. (2011). Tumor suppressor CHK2: regulator of DNA damage response and mediator of chromosomal stability. Clin. Cancer Res. 17, 401–405. 10.1158/1078-0432.CCR-10-1215 21088254

[B87] Sullivan K. A. Edmed S. L. Allan A. C. Karlsson L. J. Smith S. S. (2015). Characterizing self-reported sleep disturbance after mild traumatic brain injury. J. Neurotrauma 32, 474–486. 10.1089/neu.2013.3284 25275933 PMC4376482

[B88] Teasdale G. Jennett B. (1974). Assessment of coma and impaired consciousness. A practical scale. Lancet 2, 81–84. 10.1016/S0140-6736(74)91639-0 4136544

[B89] Tehse J. Taghibiglou C. (2019). The overlooked aspect of excitotoxicity: glutamate-independent excitotoxicity in traumatic brain injuries. Eur. J. Neurosci. 49, 1157–1170. 10.1111/ejn.14307 30554430

[B90] Thompson H. J. McCormick W. C. Kagan S. H. (2006). Traumatic brain injury in older adults: epidemiology, outcomes, and future implications. J. Am. Geriatr. Soc. 54, 1590–1595. 10.1111/j.1532-5415.2006.00894.x 17038079 PMC2367127

[B91] Tomasevic G. Laurer H. L. Mattiasson G. van Steeg H. Wieloch T. McIntosh T. K. (2012). Delayed neuromotor recovery and increased memory acquisition dysfunction following experimental brain trauma in mice lacking the DNA repair gene XPA. J. Neurosurg. 116, 1368–1378. 10.3171/2012.2.JNS11888 22462511

[B92] Tominaga T. Shimada R. Okada Y. Kawamata T. Kibayashi K. (2019). Senescence-associated-β-galactosidase staining following traumatic brain injury in the mouse cerebrum. PLoS ONE 14:e0213673. 10.1371/journal.pone.0213673 30856215 PMC6411151

[B93] Tse K. H. Herrup K. (2017). DNA damage in the oligodendrocyte lineage and its role in brain aging. Mech. Ageing Dev. 161, 37–50. 10.1016/j.mad.2016.05.006 27235538 PMC5124419

[B94] Wäljas M. Iverson G. L. Lange R. T. Hakulinen U. Dastidar P. Huhtala H.. (2015). A prospective biopsychosocial study of the persistent post-concussion symptoms following mild traumatic brain injury. J. Neurotrauma 32, 534–547. 10.1089/neu.2014.3339 25363626

[B95] Walton C. C. Begelman D. Nguyen W. Andersen J. K. (2020). Senescence as an amyloid cascade: the amyloid senescence hypothesis. Front. Cell. Neurosci. 14:129. 10.3389/fncel.2020.00129 32508595 PMC7248249

[B96] Wang J. Xiong S. Xie C. Markesbery W. R. Lovell M. A. (2005). Increased oxidative damage in nuclear and mitochondrial DNA in Alzheimer's disease. J. Neurochem. 93, 953–962. 10.1111/j.1471-4159.2005.03053.x 15857398

[B97] Wang Y. Arun P. Wei Y. Oguntayo S. Gharavi R. Valiyaveettil M.. (2014). Repeated blast exposures cause brain DNA fragmentation in mice. J. Neurotrauma 31, 498–504. 10.1089/neu.2013.3074 24074345

[B98] Wei Z. Chen X. C. Song Y. Pan X. D. Dai X. M. Zhang J.. (2016). Amyloid β protein aggravates neuronal senescence and cognitive deficits in 5XFAD mouse model of Alzheimer's disease. Chin. Med. J. 129, 1835–1844. 10.4103/0366-6999.186646 27453234 PMC4976573

[B99] Woodcock T. Morganti-Kossmann M. C. (2013). The role of markers of inflammation in traumatic brain injury. Front. Neurol. 4:18. 10.3389/fneur.2013.00018 23459929 PMC3586682

[B100] Wu D. Liu B. Yin J. Xu T. Zhao S. Xu Q.. (2017). Detection of 8-hydroxydeoxyguanosine (8-OHdG) as a biomarker of oxidative damage in peripheral leukocyte DNA by UHPLC-MS/MS. J. Chromatogr. B Analyt. Technol. Biomed. Life Sci. 1064, 1–6. 10.1016/j.jchromb.2017.08.033 28886477

[B101] Yosef R. Pilpel N. Papismadov N. Gal H. Ovadya Y. Vadai E.. (2017). p21 maintains senescent cell viability under persistent DNA damage response by restraining JNK and caspase signaling. EMBO J. 36, 2280–2295. 10.15252/embj.201695553 28607003 PMC5538795

[B102] Zhang P. Kishimoto Y. Grammatikakis I. Gottimukkala K. Cutler R. G. Zhang S.. (2019). Senolytic therapy alleviates Aβ-associated oligodendrocyte progenitor cell senescence and cognitive deficits in an Alzheimer's disease model. Nat. Neurosci. 22, 719–728. 10.1038/s41593-019-0372-9 30936558 PMC6605052

[B103] Zhang X. Chen J. Graham S. H. Du L. Kochanek P. M. Draviam R.. (2002). Intranuclear localization of apoptosis-inducing factor (AIF) and large scale DNA fragmentation after traumatic brain injury in rats and in neuronal cultures exposed to peroxynitrite. J. Neurochem. 82, 181–191. 10.1046/j.1471-4159.2002.00975.x 12091479

